# Study on the Coupling Mechanism of the Orthogonal Dipoles with Surface Plasmon in Green LED by Cathodoluminescence

**DOI:** 10.3390/nano8040244

**Published:** 2018-04-16

**Authors:** Yulong Feng, Zhizhong Chen, Shuang Jiang, Chengcheng Li, Yifan Chen, Jinglin Zhan, Yiyong Chen, Jingxin Nie, Fei Jiao, Xiangning Kang, Shunfeng Li, Tongjun Yu, Guoyi Zhang, Bo Shen

**Affiliations:** 1 State Key Laboratory for Artificial Microstructure and Mesoscopic Physics, School of Physics, Peking University, Beijing 100871, China; fengyulong@pku.edu.cn (Y.F.); jiangshuang@pku.edu.cn (S.J.); 1501110124@pku.edu.cn (C.L.); 1501110129@pku.edu.cn (Y.C.); 1601110180@pku.edu.cn (J.Z.); chenyiyong@pku.edu.cn (Y.C.); niejingxin@pku.edu.cn (J.N.); fjiao@pku.edu.cn (F.J.); xnkang@pku.edu.cn (X.K.); tongjun@pku.edu.cn (T.Y.); gyzhang@pku.edu.cn (G.Z.); bshen@pku.edu.cn (B.S.); 2 Dongguan Institute of Optoelectronics, Peking University, Guangdong, Dongguan 523808, China; shunfengli@gmail.com; 3 State Key Laboratory of Nuclear Physics and Technology, School of Physics, Peking University, Beijing 100871, China

**Keywords:** localized surface plasmon, green LED, cathodoluminescence, FDTD

## Abstract

We analyzed the coupling behavior between the localized surface plasmon (LSP) and quantum wells (QWs) using cathodoluminescence (CL) in a green light-emitting diodes (LED) with Ag nanoparticles (NPs) filled in photonic crystal (PhC) holes. Photoluminescence (PL) suppression and CL enhancement were obtained for the same green LED sample with the Ag NP array. Time-resolved PL (TRPL) results indicate strong coupling between the LSP and the QWs. Three-dimensional (3D) finite difference time domain (FDTD) simulation was performed using a three-body model consisting of two orthogonal dipoles and a single Ag NP. The LSP–QWs coupling effect was separated from the electron-beam (e-beam)–LSP–QW system by linear approximation. The energy dissipation was significantly reduced by the z-dipole introduction under the e-beam excitation. In this paper, the coupling mechanism is discussed and a novel emission structure is proposed.

## 1. Introduction

Despite GaN-based blue light-emitting diodes (LEDs) achieving rather high external quantum efficiency (EQE), the green gap is still a key issue for high-quality illumination [1,2]. Because of the imperfect structure and high polarization field in high-indium content InGaN quantum wells (QWs), the EQE for a green LED is quite low compared with that for a blue LED. Various approaches have been applied to solve this problem, such as the use of nonpolar/semipolar substrates, the use of a Si substrate, band engineering, and the introduction of a surface plasmon (SP) [3,4,5,6]. The polarization reduction and incorporation of indium into the InGaN alloy have been improved by these methods except for the last one, the SP method. Because of the high density of states (DOS) of the SP, the spontaneous emission rate (SER) in QWs can be very high, leading to a higher internal quantum efficiency (IQE) [6]. Two main perspectives on light emission enhancement via SP–QW coupling have emerged. One perspective is that the SP modes in the metal are excited by spontaneous emission (SE) in QWs and radiate to air in dipole mode [7,8], thus the energy transferred to SP is divided into “SP radiation” and “metal dissipation”. The other perspective is that the spontaneous emission rate in QWs is greatly enhanced by the strong near-field strength of the SP excited by SE in QWs [9,10,11]. Both of these perspectives can explain the light emission enhancement by SP–QW coupling. As to SP radiation, less than 50% of the coupled energy can be radiated into air, and the emission enhancement is effective for emitters with low original EQE [8]. The latter perspective introduces the possibility of emission enhancement for a green LED [10,11]. However, the energy distribution dynamics in the SP–QW coupling system remain unclear.

Although evidence of the SER enhancement has been reported for several decades, the problem of energy dissipation in the metal is not yet well resolved. The energy dissipation in the metal corresponds to the Ohmic loss and to the electron-hole pair creation [12]. Many techniques have been reported to reduce the energy dissipation effects, including using a localized surface plasmon (LSP), placing the emitters into a metal gap, adjusting the dipole orientation, and coupling the QWs between each other [8,10,11,13,14]. An enhancement of the SER exceeding 1000 times with a quantum efficiency above 50% indicates that the energy dissipation is greatly reduced by the gap modes [10]. However, the complicated fabrication procedure for the gap structure prevents its further application. LSP–QW coupling between the radial or orbital dipoles and Ag NPs is quite different in the simulation [13]. The enhancements of radiated power of a radial and an orbital dipole are induced through coupling with the lower-order (dipole) and higher-order LSP resonance, respectively. Because the conventional InGaN QWs are thin and flat, the dipoles are mainly orbitally oriented [9,13], that is, some energy dissipation always occurs for planar QW structure.

Recently, several reports of electron beam (e-beam)-excited SP in metal nanoparticles (NPs) by cathodoluminescence (CL) have been reported [15,16,17,18]. Because CL measurements combine the ultrahigh spatial resolution of an electron microscope with broadband optical sensitivity, they can be used to study the optical process in metal NPs. There is a direct link between CL and radiative modes (“bright modes”) or the radiative electromagnetic local density of states (LDOS) [16]. Some simulation results for CL measurements have also been performed by regarding the e-beam as a dipole source along the incident direction [15,18]. Although Ag NP–QW structures excited by an e-beam have been reported [19,20,21], these works did not consider the SPs induced by the e-beam. In fact, the e-beam, approximated by a vertical dipole induces the high near-field strength near the Ag NPs [15,16,17,18], will greatly influence the coupling between the LSP and the QWs. Moreover, the dipoles representing the QW and the e-beam are in-plane and out-plane, respectively. The configuration of a single Ag NP and two orthogonal dipoles can be used to study the energy transfer process of the LSP–QW coupled system.

In this work, we fabricated LSP–QW coupled samples of an array of Ag NPs embedded in photonic crystal (PhC) holes in the p-GaN layer of a green LED. Photoluminescence (PL) and time-resolved photoluminescence (TRPL) and CL measurements were carried out. A novel three-dimensional (3D) finite difference time domain (FDTD) numerical simulation model for CL and PL measurements was also put forward using Lumerical software (FDTD Solutions v8.17, Vancouver, BC, Canada) to illustrate the difference in the LSP–QW coupling mechanism under light and e-beam excitations [22]. The LSP-two orthogonal dipoles coupling mechanism is discussed and an effective way to reduce the energy dissipation in Ag NPs is proposed.

## 2. Experimental

**Figure 1:**
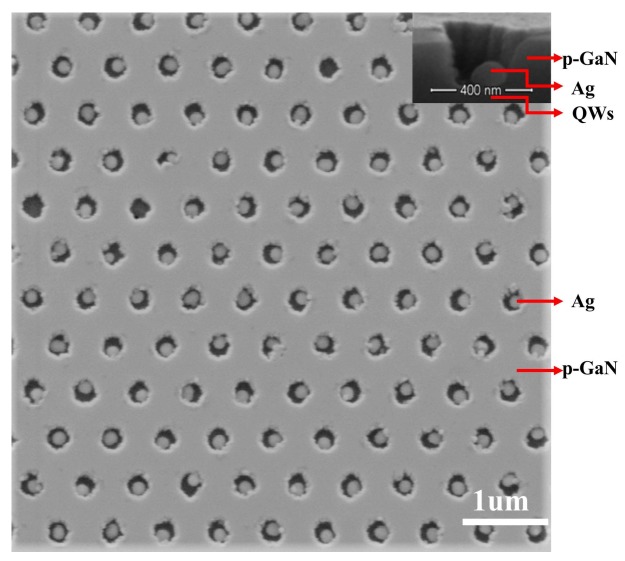
SEM image of a Ag–photonic crystal (PhC) sample. The period of the PhC is 545 nm. The diameter and height of the Ag NPs are 160 ± 10 and ~80 nm, respectively. The inset shows the cross-sectional image of a single Ag NP in the hole.

GaN-based green LED structures with a peak wavelength of 545 nm were grown by metal organic chemical vapor deposition (MOCVD) on the double-polished c-plane sapphire substrate. The structure consisted of 10 pairs of InGaN/GaN (2.5 nm/12.5 nm) multiple quantum wells (MQWs), on which is 160 nm thick p-GaN. As shown in Figure 1, LSP–QW coupled samples (Ag–PhC) were fabricated based on the PhC structure in the p-GaN layer. The scanning electron microscope (SEM) images of the Ag arrayed LED were recorded using an FEI NanoSEM 430 (FEI, Hillsboro, OR, USA). First, a 120 nm thick SiO_2_ mask was deposited onto the surface of p-GaN. The PhC patterns were obtained via nano-imprinting using an Obducat Eitre 3 instrument (Obducat, Lund, Sweden) with a standard PhC stamp whose period was 545 nm. Then, the hexagonal nanohole array was subsequently obtained using an induced coupled plasma (ICP) etcher to etch the pores to a depth of 150 nm, i.e., the bottom of each pore was 10 nm away from QWs. Then, a 30 nm thick Ag film was deposited onto the patterned surface. After thermal annealing at 600 °C for 10 min under a N_2_ atmosphere, Ag NPs were formed in the PhC holes, followed by the lift-off process to remove the Ag NPs on the SiO_2_ mask. The Ag NPs were spherical cap-shaped. Statistically, the diameter and height were 160 ± 10 and ~80 nm, respectively. For comparison, reference samples (denoted PhC) with the same pattern but without Ag NPs were also prepared.

PL measurements using a 405 nm laser diode with a power of 150 mW and a spot diameter of ~1 mm and CL measurements using a Gatan Mono-CL2 system (Gatan, Pleasanton, CA, USA) were performed at room temperature on the Ag–PhC and PhC samples with a configuration of top excitation and top detection. The electron acceleration voltage was set to 15 kV with a beam current of 158 pA. The TRPL measurements were conducted using a LifeSpec-Red picosecond lifetime spectrometer with a pulsed 372 nm laser (Edinburgh Instruments, Livingston, UK). The pulse duration was 69 ps. This instrument was made use of a time-correlated single photon counting (TCSPC) technique with a time resolution of ~30 ps within a range of 10 ns.

## 3. Results and Discussion

**Figure 2:**
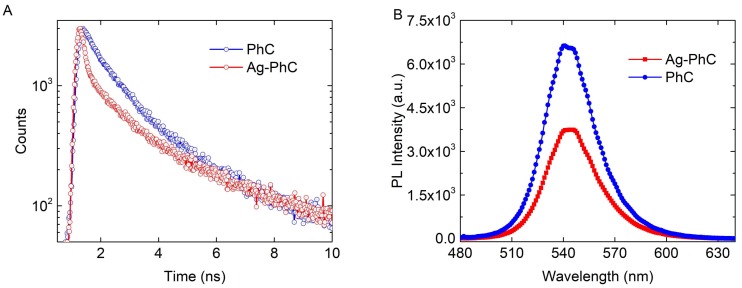
(**A**) Time-resolved photoluminescence (TRPL) and (**B**) PL spectra for Ag–PhC and PhC samples.

TRPL measurement is an efficient method to confirm LSP–QW coupling. Figure 2A shows the TRPL results at the peak wavelength 545 nm of the Ag–PhC sample and the PhC sample. The decay curves were fitted by a double exponential function as reported in the previous work [14]. The fast decay time, which corresponds to the rapid carrier recombination in InGaN QWs, were obtained as 0.23 and 0.76 ns for the Ag–PhC and PhC samples, respectively. The 3.3-fold reduction of the decay time indicates that LSP coupling with QWs substantially enhances the SER [14]. Contrarily, the PL intensity of the Ag–PhC decreased by 1.7 times compared with that of PhC sample, as shown in Figure 1B. This decrease is attributed to the large energy dissipation in Ag NPs as a result of their small aspect parameter (α), which is defined as the ratio between the height of the Ag NP and its radius [14,23]. When the α is greater than 1.5, the emission enhancement will be realized with Ag NPs with diameters ranging from 90 to 200 nm [23]. Because the LSP–QW coupled energy was either radiated into the air or dissipated in the Ag NPs, the ratio between radiated energy and the dissipated energy would determine whether the final PL intensity was enhanced or suppressed.

**Figure 3:**
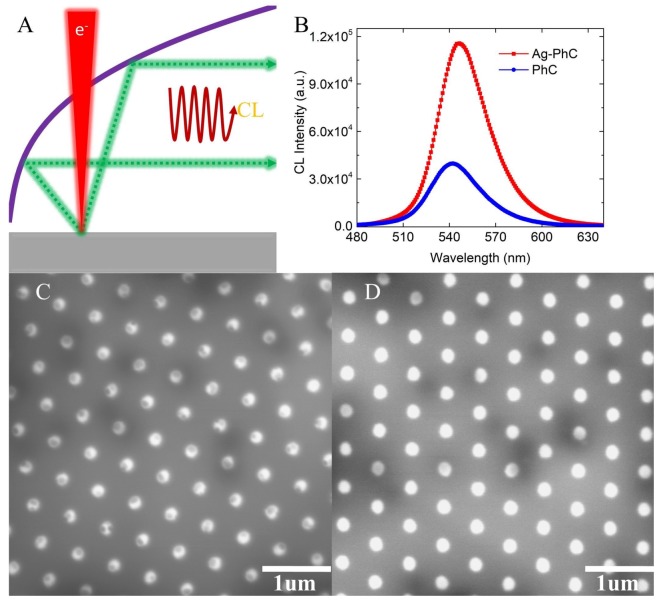
(**A**) Schematic setup for the cathodoluminescence (CL) measurement; (**B**) CL spectra for Ag–PhC and PhC samples. Panchromatic CL images for (**C**) the Ag–PhC sample and (**D**) the PhC sample. CL intensity for the Ag–PhC sample is enhanced 2.91 times compared with that for PhC sample.

A schematic setup for the CL measurement is shown in Figure 3A. An e-beam was highly focused and directed onto the surface of samples. CL measurement was performed using a 15 kV acceleration voltage and a beam current of 158 pA. The electron penetrating depth into the Ag NPs was greater than 20 nm [19,20]. The light emitted from the samples was collected by a retractable parabolic mirror and collimated to an optical monochromator, after which the signal was detected by a charge-coupled device (CCD). To ensure the maximum light collection efficiency, the samples were placed at the focal plane of the parabolic mirror, approximately ~1 mm away from the mirror. Figure 3C,D show the panchromatic CL (PanCL) image of an Ag–PhC sample and a PhC sample, respectively. The PanCL image, where all of the emitted light from the sample was collected by the e-beam scanning point by point, can clearly depict the LSP-induced luminescence around the Ag NPs. The holes are much brighter than the platform of the Ag–PhC sample. The dark spots in the holes are related to the Ag NPs. However, the spot size is much smaller than the actual size of Ag NPs in Figure 1. This discrepancy arises because of the penetration ability of high-energy electrons [19] and the strong coupling between the LSPs induced by the e-beam [16,17,18] and the QWs. The darker cloudy areas in Figure 3C,D are mainly attributed to the InGaN phase separation in QWs. CL spectra were recorded by scanning the electron beam over the entire surface of interest under the same conditions, as shown in Figure 3B. To confirm that the emission originates from QWs rather than from the Ag NPs, samples without QWs were fabricated. It was found that CL spectrum of the sample without QWs mainly originated from the emission of GaN and its intensity was approximately two orders of magnitude smaller than that of the sample with QWs. The CL intensity of the Ag–PhC sample is 2.91 times greater than that of the PhC sample, whereas the PL intensity of PhC sample is 1.7 times that of the Ag–PhC sample. Compared with laser excitation, e-beam excitation enhances the emission intensity of the Ag–PhC sample by a factor of 4.95. Considering the penetration electron energy loss in Ag NPs, even if all the electrons can penetrate through the Ag NP to excite the QW directly, it is impossible for the enhancement factor to be as high as 4.95. The high near-field strength of the LSP in the Ag NPs induced by continuously injected e-beam should be considered in the LSP–QW coupling process [16,17,18]. Moreover, how the energy dissipation is reduced by the e-beam excitation is interesting.

**Figure 4:**
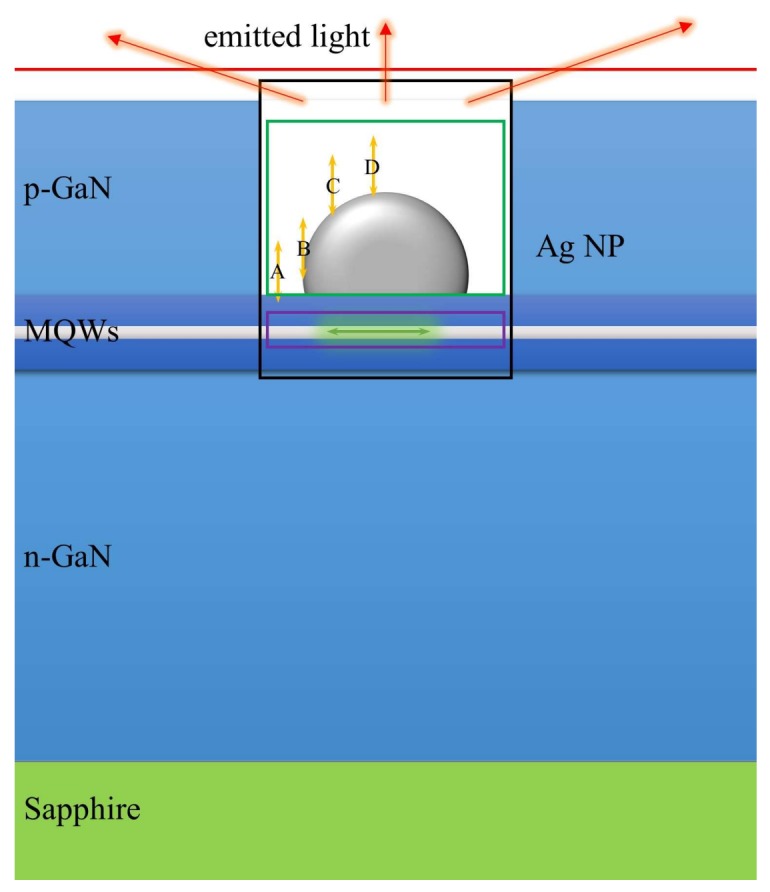
The schematic structure of the Ag–PhC sample in 3D finite difference time domain (FDTD) simulation. The purple, green, and black boxes were used to collect the total power radiated by the dipole, the dissipation power in the Ag NP, and the scatted energy, respectively. The red line (plane) was used to record the radiated power from top surface.

To distinguish the different LSP–QW coupling mechanisms by e-beam excitation and laser excitation, 3D-FDTD numerical simulations were carried out [22]. In FDTD, Maxwell’s equations are solved in discretized space and time. Figure 4 shows the schematic structure of Ag–PhC sample used in the 3D-FDTD simulation. Since the separation between Ag NPs is greater than 200 nm, the coupling between Ag NPs can be ignored [24]. Therefore, only one Ag NP needs to be considered. The dispersion relation of the Ag NP adopts “Ag (silver)-Palik (0–2 um)” data provided by the FDTD material database [22]. The Ag NP was placed in the hole center of PhC on p-GaN (*n* = 2.55) to simplify the simulation. The sizes of Ag NP and PhC are consistent with the SEM result as shown in Figure 1. The space between Ag NP and the first QW is 10 nm. The perfectly matched layer (PML) absorbing boundaries were adopted on all sides. To improve the simulation accuracy, an override region was applied over the Ag NPs and x-dipole with the mesh size of 2 nm. Outside that region, automatic graded meshing was used. Moreover, the simulation span was 6 um*6 um (x–y plane), which is large enough for the light to propagate.

Given the symmetry in the QW plane, where the radiating dipoles lie, only one dipole polarized along x direction (x-dipole) was placed below the Ag NP to represent the QW. The e-beam, however, was represented by a dipole polarized along its trajectory (z-dipole) in order to simplify the model [18]. Both the x-dipole and z-dipole adopted the built-in broadband source model, with the wavelength range from 480 to 630 nm. In FDTD, broadband sources can be used to perform simulations in which wideband frequency data is required. Since the e-beam impinging on different positions of the Ag NP may lead to different excitation, the z-dipole was successively placed with an interval of 30 nm at the positions of A, B, C, and D, as shown by the yellow arrows in Figure 4. To calculate the power transition, four monitors were used in the simulations. The purple, green, and black boxes were used to collect the power of x-dipole (QW), dissipated power, and scattering power, respectively, while the upper red plane monitor was used to record the energy successfully extracted into the air.

The actual power emitted by a dipole can vary dramatically depending on what dielectric envelopments are nearby. The field induced by a dipole or reflected from the surrounding structures can feed back on itself, causing it to radiate more or less power than expected in a homogenous material [10,11,13]. As a result, the calculated power should be normalized to the power that a dipole would emit in a homogenous material (such as vacuum), rather than the actually emitted power. In FDTD, the quantum mechanical decay rate can be related to the power collected by a monitor box in the same environment. Therefore, the decay rate can be normalized by [22],

(1) $$\frac{\gamma }{\gamma_{0}}=\frac{P}{P_{0}}$$

where *γ* and *P* are the decay rate and radiated power by a dipole in an inhomogeneous environment, and *γ*_0_ and *P*_0_ are the corresponding parameters in a homogeneous environment (here GaN). In particular, when a dipole is surrounded by a monitor box (i.e., the purple box in Figure 4), γ would become the well-known Purcell factor (*F_p_*), which is defined as the ratio of the radiation power of a dipole near Ag NPs to that in a bulk dielectric material (GaN). Thus, the EQE—namely the ratio of power measured in the far field to the total power injected into the x-dipole (QW)—as well as the IQE and light extraction efficiency (LEE) can be defined as [11,22],

(2) $$\eta_{EQE}=\eta_{IQE}\eta_{LEE}=\left(\frac{F_{p}\gamma_{rad}}{F_{p}\gamma_{rad}+\gamma_{non}}\frac{\gamma_{scat}}{\gamma_{diss}+\gamma_{scat}}\right)\frac{\gamma_{out}}{\gamma_{scat}}$$

where $\gamma_{scat}$ and $\gamma_{diss}$ are the scattering and dissipation rate, $\gamma_{rad}$ and $\gamma_{non}$ are the radiative and nonradiative decay rate, and $\gamma_{out}$ is light extraction rate. According to our temperature-dependent PL measurements, the internal quantum efficiency is calculated as 26%. A rough estimation of the ratio of the nonradiative decay rate ($\gamma_{non}$) to the radiative decay rate ($\gamma_{rad}$) for the PhC sample is 3:1. Based on the data drawn from the aforementioned monitors, EQE, IQE, LEE, and *F_p_* can be calculated, respectively.

**Figure 5:**
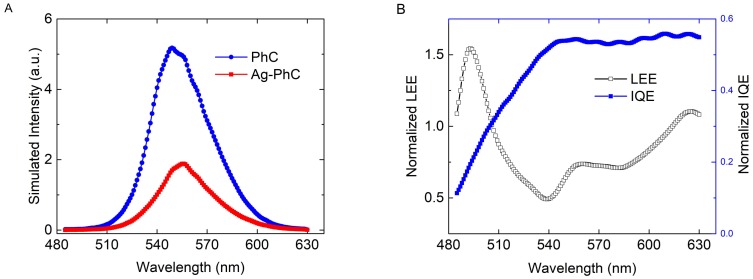
(**A**) Simulated PL spectra for Ag–PhC and PhC samples, (**B**) internal quantum efficiency (IQE) and light extraction efficiency (LEE) of Ag–PhC sample normalized to those of the PhC sample.

In the PL simulation, because the Ag NPs are opaque to light excitation [19] or the laser wavelength cannot match the SP resonant wavelength [14], z-dipole is not included, similar to our previous work [11,23]. Figure 5 shows the simulated PL spectra for the Ag–PhC and PhC samples. The IQE and LEE of the Ag–PhC sample as a function of wavelength are normalized to those of the PhC sample, as also shown in Figure 5. According to the broadband simulation results, the final emitted light spectrum can be obtained through post processing by multiplying the EQE with the actual normalized QW spectrum that is obtained without any Ag NPs nearby [11]. The simulated PL spectra show that the intensity of Ag–PhC sample decreases by 2.5 times, which agrees with the experimental result. However, the enhancement is not completely consistent with that of the experimental result, which is attributed to the approximations, including the dipole-QW approximation [9], the single Ag NP approximation [8], and the simplification of a single dipole for the QWs [11]. Moreover, the transmitted diffraction across the Ag NPs may also affect the consistence. The Purcell factor at 545 nm is calculated as 18.7, which indicates that LSP–QW coupling is strong and that the SER is greatly enhanced. However, due to the dissipation by Ag NP [11], the IQE of the Ag–PhC sample is only 54% of that of PhC sample at 545 nm. Furthermore, the LEE of Ag–PhC is also decreased by a factor of 1.8, as shown in Figure 5B. Obviously, the decreases of both IQE and LEE lead to the suppression of PL intensity.

To simulate the CL measurement for the Ag–PhC sample, the z-dipole is added to the system as shown in Figure 4. Since the e-beam excitation is dependent on its impinging position as discussed above, the simulations are carried out at e-beam impinging points A, B, C, and D individually. Considering that the electron beam itself does not radiate light, after a z-dipole is added to simulate the electron beam impinging at specific impinging points, the energy radiated by the z-dipole must be subtracted. With the presence of both the z-dipole and x-dipole, the net powers flowing through the green box, purple box, black box, and the upper red plane monitor ($P_{gBox}$, $P_{pBox}$, $P_{bBox}$ and $P_{rPlane}$ respectively) have the relationship of

(3) $$P_{rPlane}=P_{up-xDipole}+P_{up-zDipole}$$

(4) $$P_{pBox}=P_{xDipole}$$

(5) $$P_{gBox}=P_{zDipole}-\left(P_{Ag-xDipole}+P_{Ag-zDipole}\right)$$

(6) $$P_{bBox}=P_{xDipole}+P_{zDipole}-\left(P_{Ag-xDipole}+P_{Ag-zDipole}\right)$$

where $P_{xDipole}$ and $P_{zDipole}$ are the power radiated by the x-dipole and z-dipole, which can be directly recorded in the simulations. $P_{up}$ is the power extracted upward into the air, and $P_{Ag}$ is the dissipated power by Ag NP. These two quantities can also be recorded directly; however, they are the sum of the x-dipole and z-dipole components. To distinguish the efficiencies of the x-dipole (QW) from the two orthogonal dipoles system, simulations at each point without x-dipole have also been performed. Similarly, Equations (3)–(6) without x-dipole component is rewritten as

(7) $$P_{rPlane}^{\prime }=P_{up-xDipole}^{\prime }+P_{up-zDipole}^{\prime }$$

(8) $$P_{pBox}^{\prime }=P_{xDipole}^{\prime }=0$$

(9) $$P_{gBox}^{\prime }=P_{zDipole}^{\prime }-P_{Ag-zDipole}^{\prime }$$

(10) $$P_{bBox}^{\prime }=P_{xDipole}^{\prime }+P_{zDipole}^{\prime }-P_{Ag-zDipole}^{\prime }$$

where the prime (′) indicates all the powers recorded in the case without the x-dipole.

**Figure 6:**
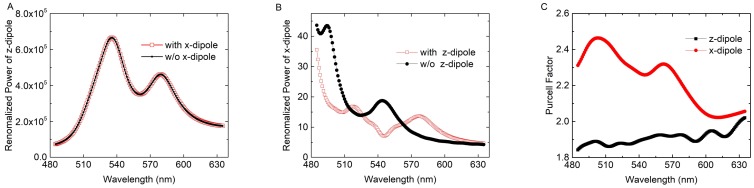
Renormalized powers vs. wavelengths of (**A**) z-dipole with and without x-dipole and (**B**) x-dipole (i.e., *Fp* in this case) with and without z-dipole at impinging point B; (**C**) Purcell factor for the x-dipole and z-dipole without the Ag NP.

As mentioned above, for the laser beam with a power of 150 mW and a spot diameter of ~1 mm, the power density of the laser beam is on the order of ~100 mW/mm^2^. Additionally, for the e-beam with a beam current of 158 pA at 15 kV impinging on the 160 nm diameter Ag NP, the power density of the e-beam is on the order of ~100 W/mm^2^. Considering the dipole-QW and dipole-e-beam approximations and that the energy of e-beam cannot be fully converted to the QW radiation, the ratio of the amplitude of z-dipole to that of x-dipole was roughly set to be only 10 since the power of a dipole is proportional to the square of its amplitude. By default, the power recorded by different detectors in Equations (3)–(6) and (7)–(10) are normalized to the sum of power from all sources ($P_{source}$). For consistency, all power in Equations (3)–(10) were renormalized by multiplying a correction factor of $P_{source}/P_{0}$, where $P_{0}$ is defined in Equation (1). Figure 6A clearly shows that $P_{zDipole}$ and $P_{zDipole}^{\prime }$ vs. wavelength almost coincide in the wavelength range from 480 nm to 630 nm at point B. We set $P_{zDipole}=\left(1+\beta \right)P_{zDipole}^{\prime }$, where β is a modification coefficient for the x-diploe. According to the simulation results, β is a small quantity. On the contrary, the power of x-dipole ($P_{xDipole}$) changes greatly with the presence of the z-dipole, as shown in Figure 6B. Figure 6C shows the Purcell factor for the x-dipole and z-dipole without the Ag NP. It is noted that the direct interaction between x-dipole and z-dipole is far weaker than that shown in Figure 6B. Therefore, the great change in Figure 6B is attributed to the SP strongly excited by z-dipole rather than z-dipole itself. Besides, the peak on the left of 545 nm in Figure 6B is enhanced with the presence of the z-dipole, indicating that high-order modes in LSP are excited and radiated [13]. According to the simulation, the electric field mapping under the Ag NP also exhibits a quadrupole characteristic at shorter wavelength peak, which again confirms the excitation of the high-order modes.

Table 1 lists the powers of z-dipole and x-dipole at different positions at 545 nm for Ag–PhC sample. The simulation results at all the points show that the presence of x-dipole can hardly affect z-dipole whereas the z-dipole strongly influences on the x-dipole through the excitation of the LSP. Thus, it is reasonable to assume that the dissipated power and extracted power of z-dipole linearly changes with $P_{zDipole}$, that is

(11) $$P_{Ag-zDipole}=\left(1+\beta \right)P_{Ag-zDipole}^{\prime }$$

(12) $$P_{up-zDipole}=\left(1+\beta \right)P_{up-zDipole}^{\prime }$$

By subtracting Equations (4)–(10) from Equations (3)–(6), the effects of x-dipole can be separated from the e-beam–LSP–QW coupling system as follows

(13) $$P_{xDipole}=P_{pBox}$$

(14) $$P_{Ag-xDipole}=P_{gBox}-\left(1+\beta \right)P_{gBox}^{\prime }$$

(15) $$P_{up-xDipole}=P_{rPlane}-\left(1+\beta \right)P_{rPlane}^{\prime }$$

**Figure 7:**
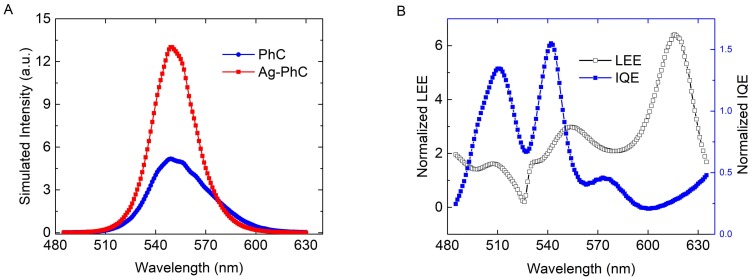
(**A**) Simulated CL spectra for Ag–PhC and PhC samples; (**B**) IQE and LEE of x-dipole normalized to those of the PhC sample at impinging point B for the Ag–PhC sample.

Based on Equations (1), (2), and (13)–(15), the EQE for the x-dipole (QW) in the e-beam–LSP–QW system can be obtained. The typical impinging positions A, B, C and D in the Ag–PhC sample are calculated and their weights are considered. The efficiencies of the x-dipole at 545 nm are also listed in the Table 1. Figure 7A shows the averaged CL spectra of Ag–PhC sample. The CL intensity of the Ag–PhC is enhanced by 2.4 times compared with that of the PhC sample, which agrees with the experimental result. The incomplete consistency is attributed to the approximations, as mentioned above. It is found that the EQE of the x-dipole in most areas around the Ag NP are enhanced by more than 2 times, except at its central area. The EQE at position D is only 0.27 times that of the PhC sample, which agrees with the panchromatic CL images in Figure 3C. In order to better understand the CL enhancement, the EQE at point B was divided to IQE and LEE, as shown in Figure 7B. Both the LEE and the IQE of the x-dipole are enhanced in the emission range compared with those of the PhC sample, which is quite different from that of the PL case. The LEE and IQE is enhanced by 2.41 and 1.43 times at 545 nm, respectively. It is notable that the *F_p_* of x-dipole at point B is the smallest in the four points in the CL simulation, as shown in Table 1. When the *F_p_* is enhanced to a certain value, it is no longer important for IQE enhancement. Since the term $\frac{F_{p}\gamma_{rad}}{F_{p}\gamma_{rad}+\gamma_{non}}$ in Equation (2) approaches 1, IQE is dominated by the scattering ratio (i.e., $\frac{\gamma_{scat}}{\gamma_{diss}+\gamma_{scat}}$ ). Compared with the simulated PL results, the scattering ratio of the x-dipole is enhanced 2.65 times. That is, when the z-dipole is added, less dissipation in the Ag NP can be obtained even when the *F_p_* decreases. According to References [8,13], with the presence of the z-dipole, the blue-shifted resonant peak in Figure 6B and the enhanced IQE in Figure 7B indicates that the original higher-order nonradiating modes and lower-order radiating modes are suppressed and enhanced, respectively. A high *F_p_* may lead to severe luminescence quenching effect via the high-order LSP modes [8].

In addition, the LEE in CL simulation is also surprisingly enhanced by 4.3 times at 545 nm at point B compared with the PL results in Figure 5B. The LEE enhancement of 68% of the Ag–PhC sample compared with the PhC sample has been obtained in our previous work, which was explained by the resultant modification of the waveguide mode, which combines the effects of the LSP and the PhC [11]. In this work, the enhancement may occur because the excessive LSP modes excited by the z-dipole also modify the waveguide mode in the GaN LEDs. The penetration ability of high-energy electrons is stronger than that of the laser, which will enhance the CL intensity as well.

As described above, the z-dipole introduction enhances both the IQE and LEE of the QWs in the LSP–QWs coupling process. The dissipated energy is reduced and the waveguide modes are extracted effectively. It is reasonable to make an orthogonal emission dipole in LSP–QW coupling systems to enhance the light output of the devices. The InGaN QWs epitaxy on pyramids or V-pit structures seems to provide the possibility of two orthogonal dipoles coupling to LSP [25], where dipole radiators within the quantum wells on the facets are similar with the z-dipole in this work compared with the dipole radiators within normal quantum wells.

## 4. Conclusions

In summary, PL and CL measurements were performed on a green LED with Ag NPs filled in photonic crystal holes. The suppression of PL and the enhancement of CL were observed compared with the PhC sample. In the FDTD simulations, the two orthogonal dipoles were used to couple with the LSP. The x-dipole (QW) effect was separated in the e-beam–LSP–QW system by linear approximation. The simulation showed that both the IQE and the LEE were enhanced by the z-dipole added to the LSP–QW system. The enhancements were attributed to the LSP excited by z-dipole coupled to some LSP modes excited by the x-dipole. A novel LED device was proposed with orthogonal emission dipoles based on the simulation and experimental results.

## Figures and Tables

**Table 1:** Powers of z-dipole and x-dipole and efficiencies of x-dipole at different positions at 545 nm for Ag–PhC sample.

Position	Power of z-Dipole		Power (*Fp*) of x-Dipole		Efficiency of x-Dipole		
	With x-Dipole	w/o x-Dipole	With z-Dipole	w/o z-Dipole	LEE	IQE	EQE
A	189.04	196.55	11.38	18.7	2.69	1.15	3.02
B	530115	530081	7.11	18.7	2.41	1.43	3.36
C	1346570	1346580	15.32	18.7	1.86	1.52	2.75
D	44715.1	44427.7	17.12	18.7	0.64	0.43	0.27
